# Mr. A. N. Ruddock’s Case of Penetrating Wounds of Both Lungs—Recovery

**Published:** 1842-06-25

**Authors:** 


*Penetrating Wounds of both Lungs—Recovery.*

[In the Provincial Medical and Surgical Journal for April 2d, 1842, Mr. A. N. Ruddock of Bristol gives the following interesting case of a police officer, aged 22 years, stabbed in both sides of the chest by a robber. It is intended to illustrate the good effects of fearless venesection.]

I saw him between five and six o'clock ; he was tolerably collected, but in a state of extreme exhaustion, from the combined circumstances of the severe nature of his wounds and the consequent loss of blood, which I found to have been considerable, and from the desperate struggle in which he had been engaged; a considerable quantity of frothy blood was passing from his mouth; he had great difficulty of breathing, with constant cough, and a very small pulse varying from 140 to 150.

On examining the chest on the right side, I found a penetrating wound of rather more than an inch in width between the seventh and eighth ribs, about four or five inches below, and in a direct line from the axilla; air was passing freely through the wound, and there was considerable emphysema. On the left side there was a corresponding wound, also, between the seventh and eighth ribs, but higher up and more posterior, situate almost in a direct line from the inferior angle of the scapula; very little air had escaped into the cellular membrane, but on passing the hand over the latissimus dorsi, the air was felt crackling under a considerable portion of that muscle. No doubt could exist as to the lungs being wounded on both sides, and I considered the prognosis as unfavourable as it could well be. As there was no fracture of the ribs, and the wounds were clean, I closed them both with adhesive plaster, applied warmth to the extremities, as he was shivering with cold, and administered some tea.

By nine o'clock, A. M., reaction had taken place; at ten o'clock his pulse had become tolerably steady at 120. Venesection to twenty ounces, which produced faintness; ordered a mixture of spermaceti and ipecacuanha wine to be taken once in three or four hours; to take nothing of any kind except tea and toast and water.

At three, P. M., he was labouring under more severe symptoms; his breathing was much oppressed; he had a good deal of pain and uneasiness about his chest, with a full pulse. Venesection to thirty-four ounces, which made him faint, and on recovering he expressed himself as much relieved.

At nine in the evening his symptoms were again aggravated, but not to so great an extent; his pulse had acquired so much firmness and strength that another blood-letting was readily foreseen, if not then absolutely necessary, and I took thirty ounces before he was faint; I ordered him ten grains of opium and soap pill, and left him for the night, with strict orders to be called if the symptoms returned.

I saw him on Friday morning at six o'clock; he had passed a tolerable night, and his state presented no symptoms which demanded interference. As I was desirous of obtaining some information about the wounds of the lungs, I removed one strip of plaster, and found that the air was passing with much less freedom than twenty-four hours ago, although the external wounds, in consequence of the continued oozing of blood, were not healing by the first intention. The wounds were again immediately closed; one ounce of castor oil was administered. Mr. R. Smith, the senior surgeon of the infirmary, saw him to day; he considered he would have a hard struggle for it, and recommended a small abstraction of blood in the evening.

Eight, P. M. Has continued up to this time pretty much as in the morning; bowels relieved. I bled him to ten ounces; repeated the opium pill.

Saturday morning. He has passed a tolerable night again, and is free from pain; pulse soft, but quick. He takes the spermaceti mixture, from which he says he finds much relief and comfort. No air whatever passes through the wounds.

Sunday morning. He remains in a quiet state; the giant has evidently been conquered since Thursday evening. The external wounds are not at all disposed to heal, but those of the lungs are apparently closed. Allowed a small teacupful of thin bread and milk, which is the first food he has taken.

From this time he slowly but gradually progressed, and he left his bed about three weeks after the injury. The bloody expectoration continued for five or six days, and the emphysema gradually subsided. The wound on the right side of the chest did not heal for a month. Adhesion of the pleura on the right side has taken place to a considerable extent, covering a space as large as the hand; on the left side the state of the part is not so easily ascertained, owing to the thick layer of muscle.

He subsequently was blistered, and took iodine for some time. He remains in the force on *reserve* duty, which does not expose him to cold or wet. He has had several attacks of shortness of breathing and pain in the side, but they have all given way to a day's confinement in bed, and a mixture of tartar emetic. These attacks have latterly been less frequent. He has got married, and at the time of my writing this he tells me his health has much improved within the last twelvemonth.

